# Activation of EGFR-PI3K-AKT signaling is required for *Mycoplasma hyorhinis*-promoted gastric cancer cell migration

**DOI:** 10.1186/s12935-014-0135-3

**Published:** 2014-12-05

**Authors:** Hongying Duan, Like Qu, Chengchao Shou

**Affiliations:** Department of Biochemistry and Molecular Biology, Peking University Cancer Hospital & Institute, Key Laboratory of Carcinogenesis and Translational Research (Ministry of Education), 52 Fucheng Road, Beijing, 100142 China

**Keywords:** *Mycoplasma hyorhinis*, Gastric cancer, p37, PI3K-AKT, EGFR

## Abstract

Persistent infection of *Mycoplasma hyorhinis* (*M. hyorhinis*) was associated with gastric cancer cell migration and invasion, but the mechanisms were not well understood. Herein, we found that *M. hyorhinis* activated phosphoinositide 3-kinase (PI3K)-AKT signaling axis in gastric cancer cell lines. Epidermal growth factor receptor (EGFR) was upstream of PI3K-AKT signaling in the context of *M. hyorhinis* infection, because phosphorylation of AKT Serine 473 was almost completely attenuated by the EGFR inhibitor AG1478 or by EGFR knockdown. Phosphorylation of AKT S473 induced by *M. hyorhinis* infection was also abolished by PI3K inhibitor wortmannin. Furthermore, we found that p37, a membrane protein of *M. hyorhinis*, could also promote *M. hyorhinis*-induced PI3K-AKT signaling activation and cell migration. In addition, pre-treatment with AG1478 or wortmannin significantly inhibited cell migration induced by *M. hyorhinis* infection or p37 treatment. In conclusion, EGFR-PI3K-AKT signaling plays an important role in *M. hyorhinis*-promoted cell migration in gastric cancer cells, thus providing a clue to the pathogenesis of *M. hyorhinis* in gastric cancer.

## Introduction

Infectious agents, such as viruses, bacteria, and parasites, have been identified as risk factors for certain typess of cancer [1]. The most famous are the association of *Helicobacter pylori* with gastric cancer and that of human papillomavirus with cervical cancer [2,3]. Identifying the roles of infectious agents in carcinogenesis and cancer development will provide more efficacious methods for prevention and therapies of these malignancies.

*Mycoplasma hyorhinis* (*M. hyorhinis*) belongs to mycoplasmas (Class Mollicutes), which are small-sized, wall-free prokaryotic organisms. The first study reporting the association of mycoplasma with cancer was in the mid-1960s. This study revealed the association between mycoplasma infection and leukemia [4]. To date, there have been several lines of clinical evidence linking mycoplasma infection to different types of cancer [5-7]. As reported by Barykova et al., *M. hominis* was present at three time higher frequency in patients with prostate cancer than in those with benign prostatic hyperplasia [7]. Meanwhile, several studies including ours have reported a potential link between *M. hyorhinis* infection and cancer [8-11]. We previously examined *M. hyorhinis* infection in over 600 human tissues using a monoclonal antibody PD4 against *M. hyorhinis* lipoprotein p37, and found that 56% of gastric carcinoma and 55% of colon carcinoma cases were *M. hyorhinis*-positive, suggesting an association between *M. hyorhinis* infection and cancer [8]. Moreover, we showed that *M. hyorhinis* infection in gastric cancer tissues positively correlates with tumor metastasis [10]. The phenotypic assays revealed that *M. hyorhinis* could promote cancer cell migration and invasion in vitro and metastasis in vivo [10]. Taken together, these results support a strong link between *M. hyorhinis* infection and cancer metastasis.

p37, a lipoprotein of *M. hyorhinis*, has no homology to any human proteins [12]. Our studies revealed that p37 enhanced the invasiveness and metastasis of gastric cancer cells in vitro and in vivo [13]. Another study reported that recombinant p37 induced anaplasia and promoted migration in prostate cancer cells [9]. However, mechanisms underlying *M. hyorhinis*’ and p37’s pro-invasive capacities are unclear.

Cancer metastasis is a multi-step process that includes: 1) vascularization of the primary tumor; 2) detachment and invasion of cancer cells; 3) intravasation into lymphatic and blood vessels; 4) survival and arrest in the circulation; 5) extravasation into distant organs; and 6) colonization and growth of metastatic tumors [14]. Nearly 90% cancer-related mortality is caused by cancer metastasis [15]. The signaling pathways involved in cancer metastasis are investigated in great detail in the past decades [16]. Among these pathways, deregulation of phosphoinositide 3-kinase (PI3K)-AKT signaling axis was observed in various kinds of cancer [17]. Previous studies uncovered the central role of PI3K-AKT signaling in several cellular processes involved in cancer, including metabolism, growth, survival, and motility [17-20].

To clarify whether PI3K-AKT signaling is activated in *M. hyorhinis*-infected gastric cancer cells and its role in cell migration, we designed and performed this study. We unveiled that *M. hyorhinis* activates PI3K-AKT signaling in gastric cancer cells in an epidermal growth factor receptor (EGFR)-dependent fashion. The activated EGFR-PI3K-AKT pathway plays an important role in *M. hyorhinis*-induced cancer cell migration.

## Results

### *M. hyorhinis* binds to gastric cancer cell MGC803 in a time and dose-dependent manner

**Figure 1:**
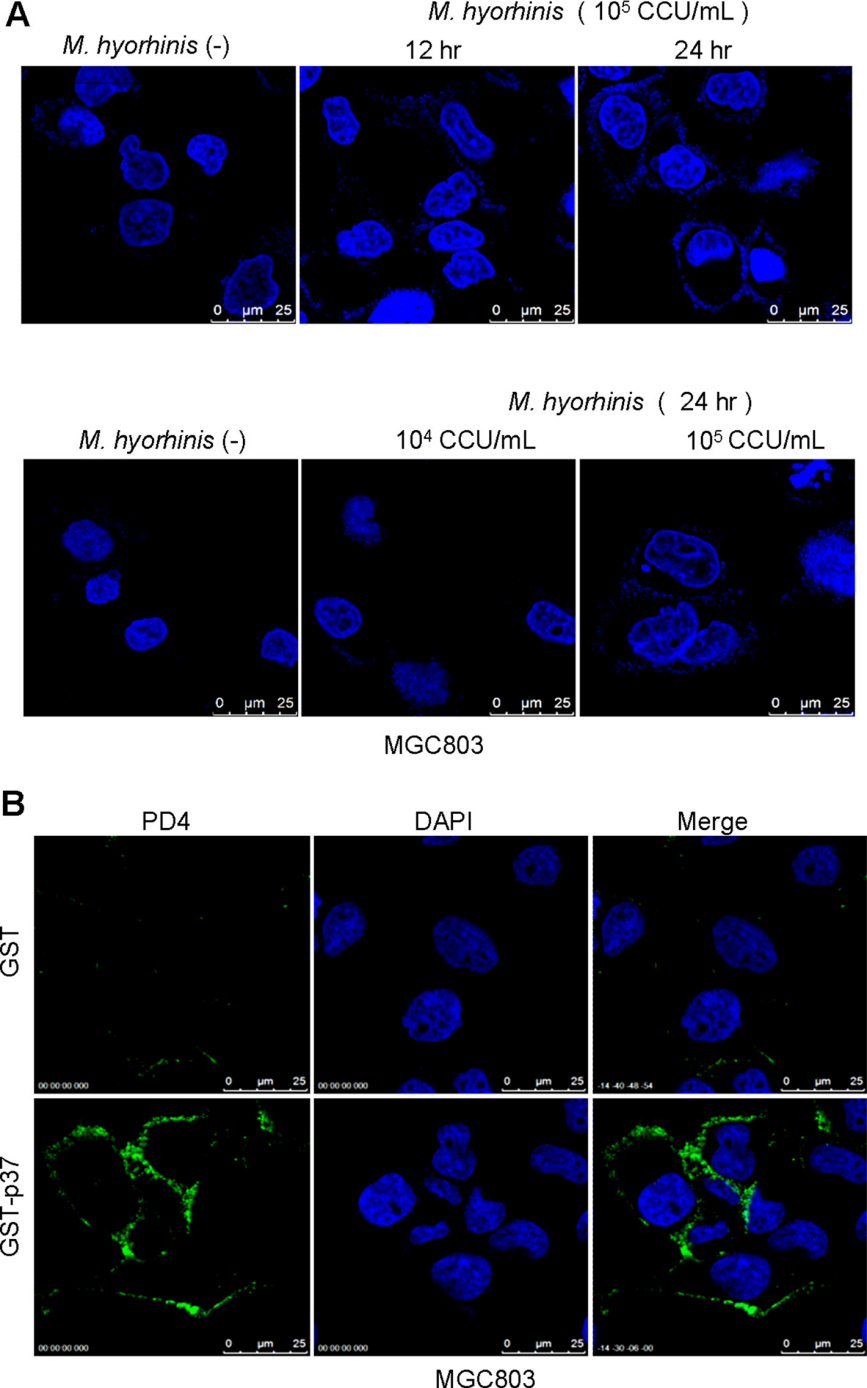
***M. hyorhinis*** **binds to gastric cancer cell MGC803 in a time and dose-dependent manner. (A)** *M. hyorhinis* binds to MGC803 cells in a time- and dose-dependent manner. The cells were exposed to 10^4^, 10^5^ CCU (color changing units)/mL of *M. hyorhinis* for 24 hours, or 10^5^ CCU/mL of *M. hyorhinis* for 0 (unexposed), 12 and 24 hours. DAPI staining was performed at indicated time points. Titer of *M. hyorhinis* was quantified as color-change-units (CCU) per milliliter. One CCU equals to one organism of mycoplasma. Unless specified, we used 10^5^CCU/mL *M. hyorhinis* to infect cells. By normalizing to the amount of cells to be infected, the multiplicity of infection (MOI) was 0.1 for 10^4^CCU/mL and 1 for 10^5^CCU/mL. **(B)** p37 binds to MGC803 cells. The cells were treated with 300 pmol GST-p37 for 24 hours. Immunofluorescence assay with PD4 antibody was performed after 24 hours. GST was used as negative control.

Our previous work has shown that *M. hyorhinis* could infect human gastric cancer cells [8,10]. Herein, through immunofluorescence staining with DAPI, we observed that *M. hyorhinis* could attach to cell membrane. *M. hyorhinis* bound to gastric cancer cell MGC803 in a time and dose-dependent manner. When 1 × 10^5^ CCU/mL *M. hyorhinis* was added in the cell culture medium and incubated with cells for 24 hours, peri-nuclear DNA staining was clearly seen by confocal microscopy immunofluorescence assay (Figure 1A). p37 protein is the most abundant membrane moiety of *M. hyorhinis* [12]. In this study, we found that recombinant GST-p37 fusion protein, but not GST, could adhere to MGC803 cell membrane, as shown by immunofluorescence staining with PD4 antibody (Figure 1B), suggesting that p37 may exert some roles in *M. hyorhinis* infection of human cells.

### Both *M. hyorhinis* and GST-p37 activate PI3K-AKT signaling

**Figure 2:**
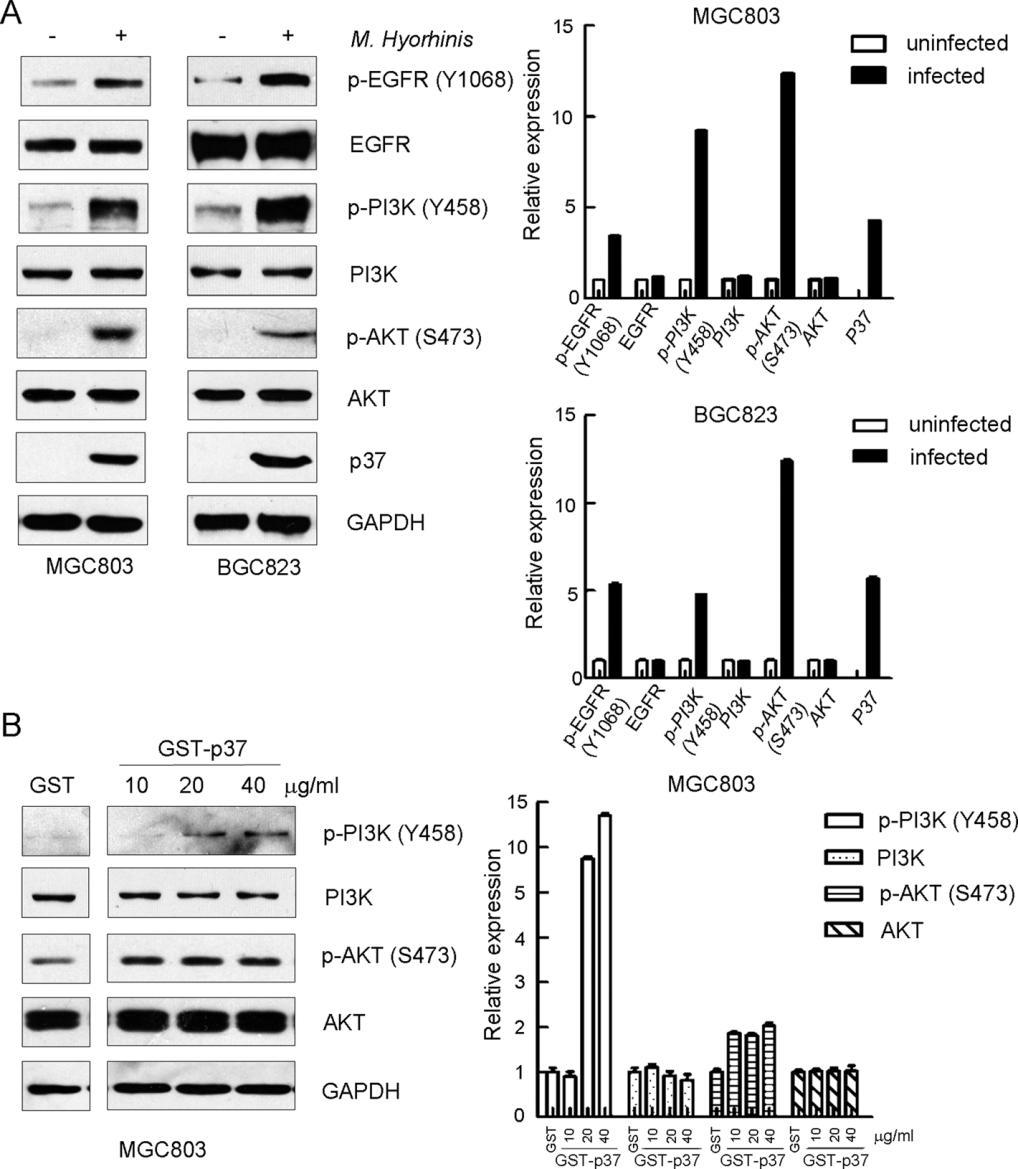
**Both** ***M. hyorhinis*** **and p37 activate PI3K-AKT signaling. (A)** *M. hyorhinis* upregulates EGFR, PI3K and AKT phosphorylations in gastric cancer cell MGC803 and BGC823. Cells were serum starved for 24 hours and then infected with *M. hyorhinis* for another 2 hours. Protein lysates were analyzed by Western blot with indicated antibodies. **(B)** Purified p37 protein upregulates PI3K and AKT phosphorylations in gastric cancer cell MGC803. Cells were serum starved for 24 hours and then treated with p37 for another 2 hours. Protein lysates were analyzed by Western blot with indicated antibodies. Optical densities of protein bands were quantified by Image J software and relative expression levels of indicated protein to loading control were shown in graph. Values represented the mean ± SD from three to four independent experiments.

We previous reported that *M. hyorhinis* could induce cancer cell migration and invasion [10]. Our study also revealed that both purified p37 protein and adenovirus-mediated overexpression of p37 could promote AGS gastric cancer cell invasiveness and metastasis [13]. PI3K-AKT signaling is deregulated in a range of human cancers and is thought to promote tumorigenesis and cancer metastasis [21]. We noticed that phosphorylations of PI3K and AKT were increased in *M. hyorhinis*-infected MGC803 and BGC823 cells, while total levels of these two proteins were stable (Figure 2A). When the cells were treated with GST-p37 in different doses for 2 hours, the PI3K and AKT were also dramatically activated (Figure 2B), suggesting that p37 alone is sufficient to induce PI3K and AKT phosphorylations upon *M. hyorhinis* infection.

### PI3K-AKT signaling is downstream of EGFR in *M. hyorhinis*-infected MGC803 cells

**Figure 3:**
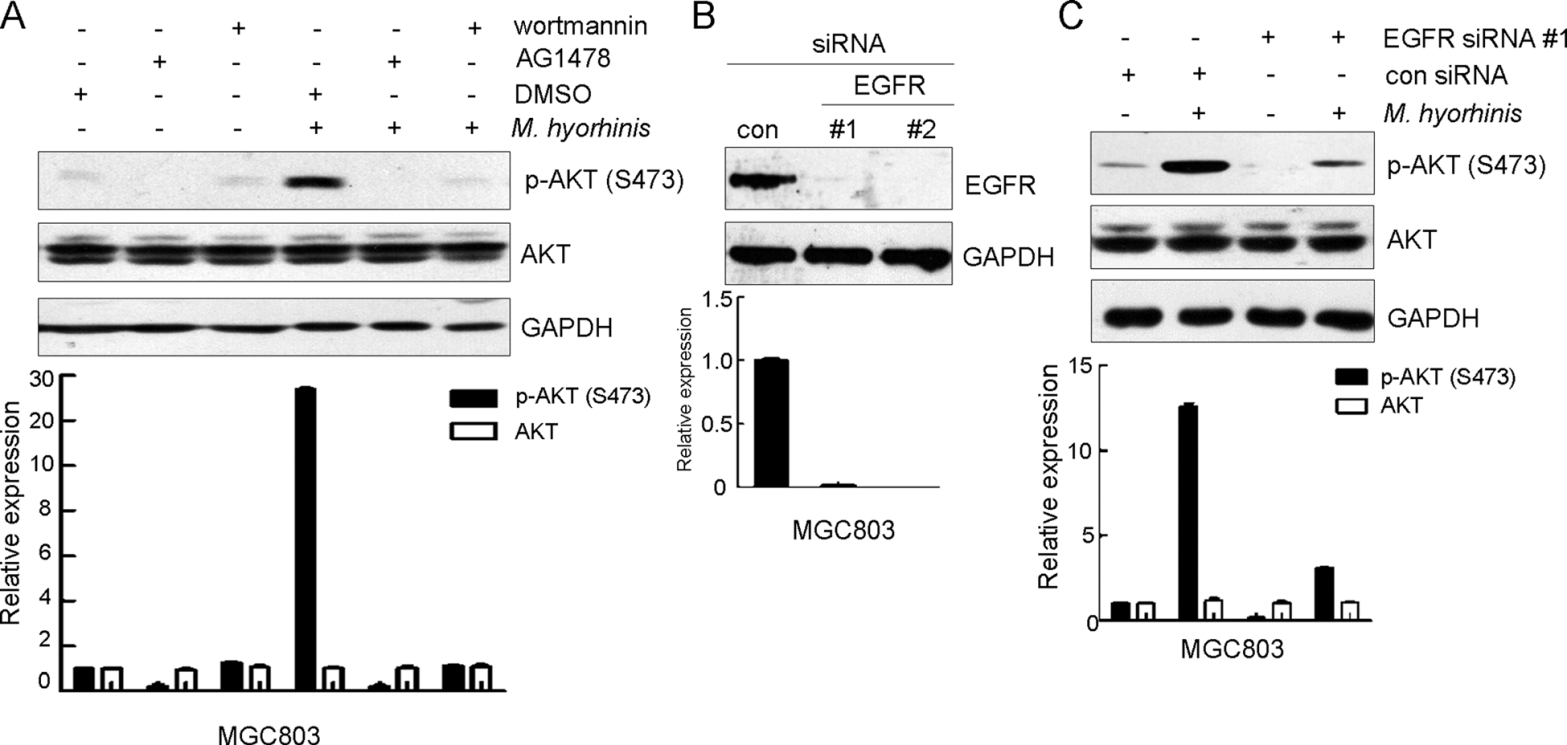
**PI3K-AKT signaling is downstream of EGFR in** ***M. hyorhinis*** **-infected MGC803 cells. (A)** AG1478 or wortmannin pre-treatment abolishes *M. hyorhinis*-induced AKT phosphorylation. Prior to *M. hyorhinis* infection, cells were pretreated with 5 μM AG1478 or 2 μM wortmannin for 1 hour. After that, Western blot were performed. **(B)** Validation of silencing efficiency of EGFR. Cells were transiently transfected with 50 nM EGFR-specific siRNAs or a control siRNA. 48 hours after transfection, cell lysates were subjected to Western blot. **(C)** Knock down of EGFR abolishes *M. hyorhinis*-induced AKT phosphorylation. Cells were transiently transfected with 50 nM specific siRNA targeting EGFR. 48 hours after transfection, cells were infected with *M. hyorhinis* for 24 hours, followed by Western blot. Optical densities of protein bands were quantified by Image J software and relative expression levels of indicated protein to loading control were shown in graph. Values represented the mean ± SD from three independent experiments.

We found that *M. hyorhinis*-induced phosphorylation of AKT S473 was attenuated by PI3K inhibitor wortmannin (Figure 3A), confirming that PI3K is upstream of AKT in the context of *M. hyorhinis* infection. PI3K-AKT signaling can be activated by multiple stimuli. Growth factor receptor family proteins belong to major upstream molecules of PI3K-AKT signaling [22]. EGFR was shown to be involved in *Helicobacter pylori*-induced activation of EGFR-PI3K signaling in AGS cells [23,24]. Interestingly, phosphorylation of EGFR Y1068 was increased in *M. hyorhinis* infected cells (Figure 2A). To explore the role of EGFR in *M. hyorhinis*-promoted PI3K-AKT signaling, we utilized EGFR kinase inhibitor AG1478 and RNA interference strategies respectively. After AG1478 pre-treatment, we found that phosphorylation of AKT S473 in *M. hyorhinis*-infected MGC803 cells was reversed to the level similar to that in non-infected counterpart (Figure 3A). Alternatively, when EGFR expression was knocked down by a specific siRNA (Figure 3B), *M. hyorhinis* infection-induced phosphorylation of AKT S473 was also counteracted (Figure 3C).

### PI3K-AKT signaling is required for *M. hyorhinis* infection and induced cell migration in MGC803 cells

**Figure 4:**
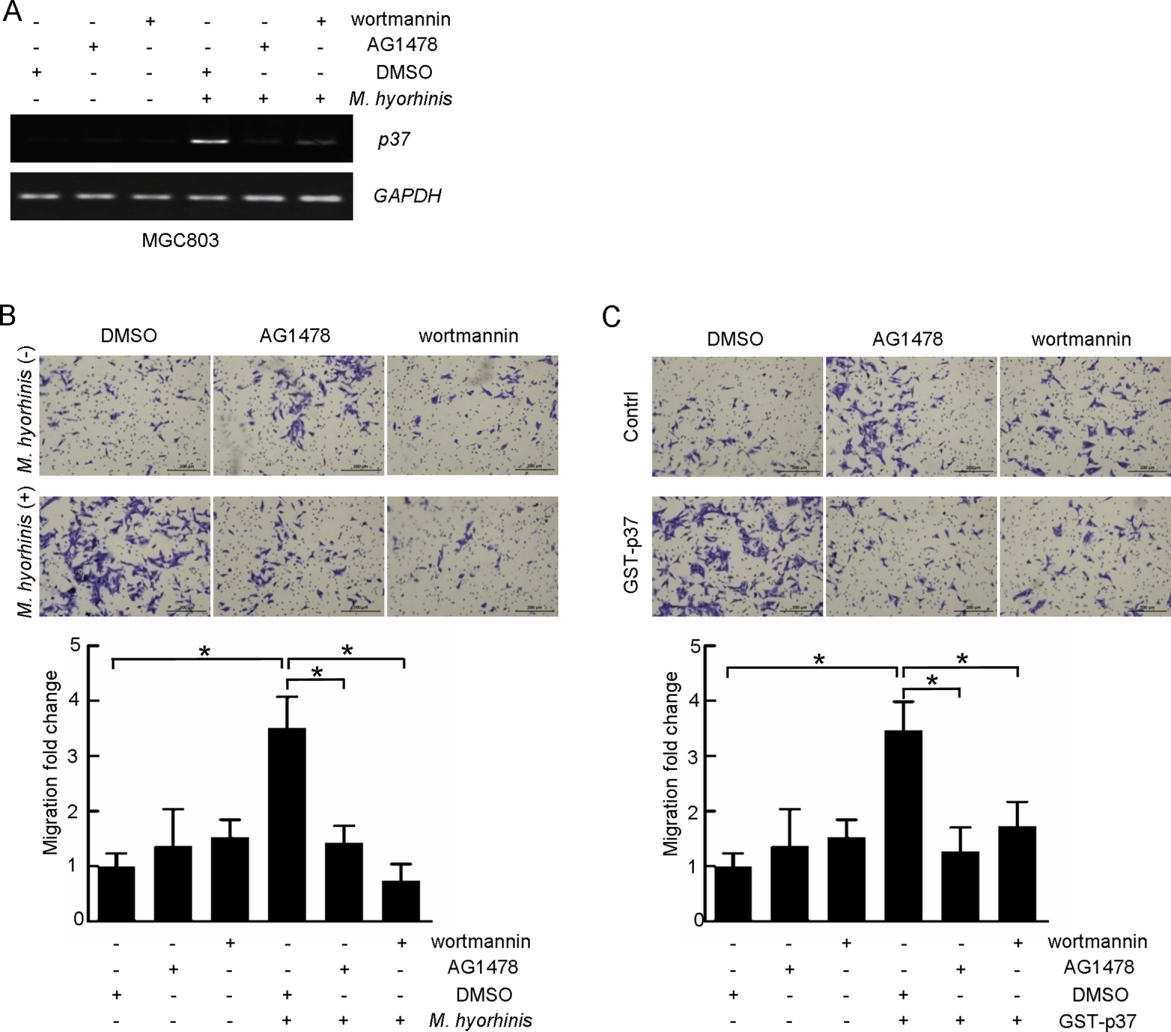
**PI3K-AKT signaling is required for** ***M. hyorhinis*** **infection and induced cell migration in MGC803 cells. (A)** AG1478 or wortmannin pretreatment inhibits *M. hyorhinis* infection. AG1478 or wortmannin was added in cell medium 1 hour prior to *M. hyorhinis* exposure. Then the cells were subjected to PCR amplification of *p37*. **(B)** and **(C)** AG1478 or wortmannin abolishes *M. hyorhinis* **(B)** and GST-p37 **(C)** induced cell migration. Transwell cell migration assay was performed according to manufacturer’s protocol. Values represented the mean ± SD from three to four independent experiments with triplicate samples. *, *P* <0.05.

Next, we sought to determine the contribution of PI3K-AKT signaling to *M. hyorhinis* infection. We found that the infection of MGC803 cells were partially blocked by AG1478 and wortmannin, as shown by lowered band intensity of *p37* in PCR assay (Figure 4A). Meanwhile, we found these two inhibitors significantly lowered *M. hyorhinis*- and p37-induced cell migration (Figure 4B and C), suggesting that activated EGFR-PI3K-AKT axis is responsible for *M. hyorhinis*- or p37-induced cell invasiveness.

## Discussion

Epidemiologic and molecular studies suggest that microbial infections are associated with certain cancers. It has been suggested that there is an association between mycoplasma infection and different cancers [25,26]. Since the anti-tumor monoclonal antibody PD4 was developed by our lab [27], we sought to identify the antigen of PD4. Surprisingly, the antigen recognized by PD4 was characterized as a lipoprotein from *M. hyorhinis*, namely p37 [28]. Immunohistochemical studies utilizing PD4 suggested a strong association of *M. hyorhinis* infection with cancer metastasis [8,10].

Most cancer-related deaths are caused by metastasis [15]. Several signaling pathways were shown to be responsible for this process [16]. Among them, PI3K-AKT signaling was investigated in great detail [17-20]. In the present study, phosphorylations of PI3K and AKT were found to be upregulated after *M. hyorhinis* infection or by treatment with recombinant p37 protein. These results suggested that PI3K-AKT signaling was activated in the process of *M. hyorhinis* infection, which might be mediated by its membrane protein p37. PI3K, consisting of a regulatory subunit p85 and a catalytic subunit p110, is often activated by growth factor stimulation through receptor tyrosine kinases (RTKs) [29-31]. The regulatory subunit, p85, directly binds to phosphotyrosine residues on RTKs. This binding relieves the intermolecular inhibition of the p110 catalytic subunit by p85 and localizes PI3K to the plasma membrane where its substrate, phosphatidylinositol 4, 5-bisphosphate, resides [30]. These RTKs are often mutated, amplified, or overexpressed in cancers, causing aberrant PI3K activation [30]. For example, PI3K is activated by EGFR in lung cancers harboring somatic activating mutations in EGFR, and by human epidermal growth factor receptor 2 (HER2) in breast cancers with HER2 amplification [29,32,33]. In the context of *M. hyorhinis* infection, EGFR was phosphorylated and activated [10]. In this study, to assess the role of EGFR in *M. hyorhinis*-induced PI3K-AKT signaling activation, both chemical inhibition of EGFR by AG1478 and knockdown of EGFR via RNAi were applied. We found that *M. hyorhinis* infection-induced phosphorylations of PI3K and AKT were dependent on EGFR, therefore *M. hyorhinis* infection could induce activation of EGFR-PI3K-AKT signaling. As to how *M. hyorhinis* infection activates EGFR, we propose that *M. hyorhinis* may bind to certain cell surface protein(s) via p37, in turn recruiting EGFR on the cell membrane and promoting EGFR homodimerization or heterodimerization with other epidermal growth factor family proteins. After dimerization, EGFR was autophosphorylated and then phosphorylated downstream signaling targets such as PI3K. In the future work, we will focus on the work searching for cellular factors mediating *M. hyorhinis* infection-induced downstream signaling events.

Activated PI3K catalyzes the synthesis of the membrane phospholipid phosphatidylinositol 3,4,5-triphosphate from phosphatidylinositol 4,5-bisphosphate, thus recruiting AKT to the plasma membrane by direct interaction of phosphatidylinositol 3,4,5-triphosphate with the AKT pleckstrin homology (PH) domain [34]. Several studies have reported increased AKT phosphorylation and protein expression in tumors of the breast, prostate, ovary, and pancreas [35-38]. Once phosphorylated, AKT relocalizes to subcellular compartments where it phosphorylates substrates to exert distinct functions, such as cell growth, survival, anti-apoptosis and cell migration [20,39,40]. Among the above-mentioned cellular processes, the major function of AKT is its role in promoting cell growth [39]. The contribution of this signaling axis to cell proliferation and survival has already been widely discussed. The classical mechanism appears to be through activation of the mammalian target of rapamycin complex 1 (mTORC1), which is regulated by both nutrients and growth factor signaling [41]. However, growth and survival are not the only phenotypes that exist in carcinomas. Additionally, cell migration and invasion are also important phenotypes that are responsible for the progression of primary tumors into metastases [20]. Pro-migratory function of PI3K-AKT signaling has been underscored by some recent studies [20,42,43]. A key discovery made by the Mercurio laboratory, showed that the alpha6beta4 integrin, a tumor-associated antigen, promoted breast and colon cancer cell migration and invasion by activating PI3K-AKT signaling [44]. Furthermore, AKT can stimulate secretion of matrix metalloproteases that are required for degradation of the extracellular matrix [45]. In fibroblasts, AKT signaling enhances activation of various small GTPases, including Rac, and thus leading to remodeling of the actin cytoskeleton and enhanced cell motility [46]. Similarly, expression of activated AKT in fibrosarcoma or pancreatic cancer cells increases their invasion through Matrigel, an effect recapitulated by overexpression of AKT in breast and ovarian cancer cells [45,47,48]. Expression of AKT can also promote epithelial-mesenchymal transition (EMT), a process intimately associated with tumor progression to invasive and metastatic carcinoma [49]. In the present study, we found that AKT is activated in *M. hyorhinis*-infected gastric cancer cells, and this activation is required for *M. hyorhinis*- and p37-induced cell migration in gastric cancer cells. Our results support the point that PI3K-AKT axis plays an important role in *M. hyorhinis* infection and *M. hyorhinis*-induced cancer cell migration. Because AKT phosphorylates different targets involved in a large variety of cellular processes, it is possible that *M. hyorhinis*-induced AKT activation might affect other aspects of cellular processes, such as anti-apoptosis, a possibility need to be validated in the future work.

Not surprisingly, the PI3K-AKT pathway has attracted great attention in attempts to develop specific inhibitors as therapy for various cancers, and numerous agents were developed [50,51]. In this study, we discovered activation of the EGFR-PI3K-AKT pathway after *M. hyorhinis* infection in gastric cancer cells, which may provide valuable evidence for patient-directed therapies of *M. hyorhinis*-positive gastric cancer patients.

## Materials and methods

### Cell culture

MGC803 gastric cancer cell line was from a 53-year-old male Chinese with poorly differentiated mucoid adenocarcinoma, while BGC823 gastric cancer cell line was from a 62-year-old male Chinese with poorly differentiated adenocarcinoma. Both cell lines were cultured in RPMI-1640 medium supplemented with 10% fetal bovine serum (FBS) and incubated in 5% CO2 at 37°C. The culture media and FBS were obtained from Invitrogen (Carlsbad, CA, US). Mycoplasma test was routinely performed by PCR amplification of *M. hyorhinis p37* and DAPI staining of mycoplasma DNA.

### Mycoplasma propagation and co-culture

*M. hyorhinis* was grown for 3–4 days at 37°C in Hayflick’s medium [52]. *M. hyorhinis* was serially passaged for 3 times and harvested by centrifugation for 20 min at 12,000 g, washed once in PBS, resuspended in PBS, and stored at −80°C. Titer of *M. hyorhinis* was quantified as color change units (CCU) per milliliter as described previously [53]. MGC803 and BGC823 cells were serum-starved for 24 hours prior to addition of *M. hyorhinis*. Cells were incubated with *M. hyorhinis* for 24 hours before experiment.

### Reagents and antibodies

GST and GST-p37 fusion proteins were expressed and purified by our lab. AG1478 was purchased from Sigma-Aldrich (St Louis, MO, US) and wortmannin was purchased from Cell Signaling (Danvers, MA, US). Both of them were dissolved in DMSO, aliquoted and stored at −20°C. Anti-phospho-EGFR (Y1068) (#2234), anti-phospho-PI3K (S458) (#4228), anti-phospho-AKT (S473) (#4060), anti-PI3K (#4257), and anti-AKT (#4691) antibodies were purchased from Cell Signaling. Anti-AKT antibody (BS1784) was purchased from Bioworld (St. Louis Park, MN, US). Anti-p37 monoclonal antibody PD4 was generated in our lab [27].

### Immunofluorescence

Cells were grown on the coverslips and fixed with 4% paraformaldehyde for 20 min at room temperature, followed by blocking with 5% bovine serum albumin at room temperature for 1 hour. The cells were incubated with PD4 overnight at 4°C, followed by washing with PBS/0.1% Tween-20 and probing with FITC-conjugated anti-mouse antibody for 45 min at room temperature in dark. After washing with PBS/0.1% Tween-20 for 3 times, the cells were counterstained with DAPI and mounted on 50% glycerol/PBS. For the staining of *M. hyorhinis* DNA, cells were directly stained with DAPI and mounted on 50% glycerol/PBS after blocking. A Leica SP2 confocal microscope (Leica Microsystems, Dresden, Germany) was used to observe the localization of p37 or *M. hyorhinis* DNA.

### Small interference RNA (RNAi) transfection

Cells were plated in 12-well plates and transfected with siRNA mate (GenePharma, Shanghai, China) according to the manufacturer’s protocol. The target sequences were as follows: EGFR #1 5’-CCACCUCUCUACCUUAAUATT-3’ , #2 5’-GGGCUUAGAACAACUAGAATT-3’. Negative control siRNA sequence was 5’-ACACGAGAUAAUAUCGACUUG-3’. siRNAs were synthesized by GenePharma (Shanghai, China).

### Western blot

Cells were washed twice with cold PBS and then lysed in lysis buffer (25 mM Tris–HCl pH 7.4, 150 mM NaCl, 1 mM EDTA, 1% NP-40, 10% glycerol, and 1× complete protease inhibitors). The proteins of the lysates were quantified with BCA^TM^ Protein Assay Kit (Thermo Fisher, Waltham, MA, US). 50 μg of total proteins were subjected to Western blot with indicated antibodies and GAPDH was used as loading control.

### DNA extraction and detection of *M. hyorhinis* by PCR

DNA was isolated from cells by digestion with 50 mM Tris pH 8.5, 1 mM EDTA, 0.5% Tween-20, and 200 mg/L proteinase K, followed by phenol/chloroform/isoamyl alcohol extraction and sodium acetate precipitation. DNA precipitates were washed with 70% ethanol, dried, and dissolved in 20 μL sterile water. PCR was performed with 0.5 μg extracted DNA and *p37*-specific primers (forward: 5’-GTAGTCAAGCAAGAGGATGT-3’, reverse: 5’-GCTGGAGTTATTATACCAGGA-3’) [54]. For PCR, the DNA was denatured at 94°C for 5 min first, followed by 30 cycles of 94°C for 30 sec, 50°C for 30 sec, and 72°C for 60 sec, then at 72°C for 10 min. The PCR products were analyzed by agarose gel electrophoresis. *GAPDH* was amplified as loading control.

### Cell migration assay

Tissue culture-treated 6.5-mm Transwell chamber with 8.0-μm pore membranes (Corning, Corning, NY, US) was used. Each well of 24-well plates was filled with 800 μL medium containing 10% FBS. 3×104 cells, containing *M. hyorhinis* plus inhibitors or DMSO, were transferred onto the top chamber of each Transwell apparatus (100 μL/chamber). Cells were incubated for 24 hours at 37°C. After that the chambers were then fixed in methanol, stained with hematoxylin, and counted in five randomly selected microscopic fields per well. Each sample was prepared in triplicate chambers and each experiment was repeated for at least 3 times.

### Statistical analysis

The data were analyzed by ANOVA. The statistical analysis was done using SPSS 11.0 software (SPSS) and *P* <0.05 was considered significantly.

### Ethical approval

No experiment was carried out on humans or animals.

## Abbreviations

CCU: Color changing units
EGFR: Epidermal growth factor receptor
EMT: Epithelial-mesenchymal transition
FBS: Fetal bovine serum
HER2: Human epidermal growth factor receptor 2
*M. hyorhinis*: Mycoplasma hyorhinis
mTORC1: mammalian target of rapamycin complex 1
PH: Pleckstrin homology
PI3K: Phosphoinositide 3-kinase
RTKs: Receptor tyrosine kinases
